# Assessment of Micronutrient Situation among Reproductive-Age Women (15–49) and Under-Five Children in Sudan

**DOI:** 10.3390/nu13082784

**Published:** 2021-08-13

**Authors:** Eiman S. elkhalifahassan Swareldhab, Ayoub Al-Jawaldeh, Abdul Baseer Qureshi, Amira M. Elmunier Ali, Mohamed Abu-Manga, Maha Al-Areeqi, Fekri Dureab

**Affiliations:** 1 Federal Ministry of Health, Khartoum 11111, Sudan; emanos56@gmail.com (E.S.e.S.);mohabumanga80@gmail.com (M.A.-M.); 2 World Health Organization (EMRO), Cairo 7608, Egypt; aljawaldeha@who.int; 3 World Health Organization (WHO), Khartoum 11111, Sudan; qureshiab@who.int (A.B.Q.); alia@who.int (A.M.E.A.); 4 Public Health Specialist, Sana’a, Yemen; maha_alareeqi@yahoo.com; 5 Heidelberg Institute of Global Health, Hospital University Heidelberg, 69115 Heidelberg, Germany; 6 Institute for Research in International Assistance, Akkon Hochschule, 12099 Berlin, Germany

**Keywords:** micronutrients, undernutrition, women of reproductive age, under-five children, Sudan

## Abstract

Background: Micronutrient malnutrition is a form of undernutrition that causes diseases, and this is mainly due to insufficient intake of nutrients in daily foods. The status of micronutrients for people in Sudan remains scarce, and information is limited. The aim of this study is to highlight the status of micronutrients among women of reproductive age (15–49 years of age) and their children in Sudan. Methods: This manuscript is a quantitative descriptive study, based on the data from Sudan Micronutrient Survey (SMS); it is part of the second round of the Simple Spatial Survey Method (S3M II) in Sudan (a total of 93,882 households). Results: The level of consumption of vitamin A-rich foods was found to be moderate at 67.36% for reproductive-age women and low at 23.44% for under-five children. Similarly, consumption rate of vitamin B-rich foods among reproductive-age women was 62.13%, and low for children at 11.02%. The consumption of iron-, calcium-, and zinc-rich foods was moderate among women (66.75%, 47.69%, 69.72%, respectively) and very low in children (12.28%, 17.62%, 14.99%, respectively). The iron deficiency prevalence was 47% in non-pregnant women, 58% in pregnant women, and 54% in children. The prevalence of anemia was 30% in non-pregnant women, 37% in pregnant women, and 48% in children. Generally, urinary iodine concentration was inadequate in lactating and non-pregnant women as well as in pregnant women. Most indicators of micronutrients in Sudan for children and women of reproductive age were highly significant. Sudan needs more efforts to create an enabling environment through legislation, policies, and strategies to strengthen the nutrition-sensitive and specific interventions and improving status of micronutrients among women and children, focusing on food fortification, food supplements, and counseling on micronutrients intake for mothers during antenatal and postnatal services as well as raising community awareness.

## 1. Introduction

Deficiency of micronutrients is the second strand of malnutrition often referred to as a hidden hunger and globally recognized as a serious health problem that deprives children of their lives [1,2]. Over two billion people worldwide are estimated to be deficient in one or more vitamins and minerals [3]. The numbers of children affected are substantial: around 340 million children under five suffer from micronutrient deficiencies [2].

Malnutrition in children may occur due to the maternal malnutrition that affects the child during pregnancy and childbirth. Maternal malnutrition, represented by underweight and anemia, increases the risk of perinatal complications and mortality, which lead to low birth weight, impaired mental development, and increased risk of chronic disease and mortality during the infancy period. Therefore, the risks of inadequate growth, stunting, wasting, and frequent infections increase in early childhood and adolescence [2,4,5].

Micronutrients malnutrition is a form of undernutrition mainly due to inadequate intake of nutrients in daily foods. Deficiencies in micronutrients include vitamins A, B-complex, D, and E, and minerals iron, folic acid, calcium, zinc copper, magnesium, selenium, and iodine [6]. Iron deficiency is the most common cause of nutritional anemia. Globally, 38% of pregnant women and 43% of children under five years of age suffer from anemia [7]. Iron deficiency anemia in pregnancy can cause low birthweight and inadequate folate concentrations in the first trimester of pregnancy, which increases the risk of neural tube defects such as spina bifida in the fetus. Calcium deficiency increases the risk of preeclampsia and premature labor. Vitamin A deficiency weakens the immune system and stymies the growth of the fetus. Zinc deficiency is associated with low birth weight, poor fetal neurodevelopment, premature birth, and increased neonatal mortality [2,7,8].

Sudan is experiencing a high rate of malnutrition among the Eastern Mediterranean Region, with global stunting prevalence of 36.35% and a global wasting prevalence of 13.6% [9]. Despite the fact that micronutrient deficiencies are common and contribute significantly to specific disease risks, population micronutrient status data remain scarce [10]. In Sudan, information about micronutrients is limited. A recent hospital-based study was conducted to determine the micronutrient dietary intake of pregnant Sudanese women [11]. Another comparative assessment of nutrient intake (macronutrients and micronutrients) of displaced and non-displaced women was carried out on a small scale in an urban community [12]. Therefore, this manuscript highlights the micronutrient status among reproductive-age women (15–49 years of age) and their children in Sudan.

## 2. Materials and Methods

This manuscript is a quantitative descriptive study based on the data from Sudan Micronutrient Survey (SMS); it is part of the second round of the Simple Spatial Survey Method (S3M II) in Sudan that was completed in 2019 [13]. In this manuscript, we used the data from the survey report to describe the micronutrient indicators in children under five years of age and women of reproductive age [14].

Dietary intake data were also assessed by estimating consumption of specific micronutrients based on food consumed in the 24 h (both day and night) prior to the survey.

### 2.1. Methods Used in S3M II-Micronutrient Survey

#### 2.1.1. Survey and Sample Design

In this manuscript, the SMS spatially stratified cluster survey was used, which was conducted in two stages; it was carried out within the comprehensive sampling scope of the S3M II. The S3M II is an area-based sampling survey. The process of its sampling was planned in a way to represent the entire country, including the small administrative units and localities. The primary sampling unites (PSUs) of the S3M II were selected, and they were the pool of selection for the PSUs of the SMS. From each state, there was a random selection of 30 PSUs which were selected from the selected PSUs of the 2018 S3M II in the same states. The second stage of sampling of the SMS was made for the completely eligible respondents (mother/caregiver, child pairs) in the households who were selected for the S3M II. The number of samples was crucial in order to end with an appropriate sample size to come up with a useful and precious estimation of the different indicators at the state and national level (a total of 93,882 households including children under the age of five represented 9703 samples and women in the reproductive age represented 8635 samples (4187 non-pregnant lactating, 1784 pregnant, 2682 non-pregnant, and non-lactating).

Blood and urine samples were collected from participants appropriately. They were stored, transported, and analyzed as required, following all guidelines. The blood samples were collected through a venipuncture, situated in a tube to be clotted, centrifuged, and then separated into the serum to be used. For the urine samples, they were collected in a sterile and single-use plastic container with a lid. All the samples from one cluster were packed in one container, transported to the nearest suitable laboratory in each state, and stored in a freezer kept at −20 °C until transport to the laboratory to be analyzed for iodine. From the 18 states of Sudan, the total blood samples were 18,356, including the two targeted groups.

The targeted sample of this survey was taken from those who met the criteria of the study. These criteria were contained within two groups based on age. The first group was children under the age of five years. The second group was women of reproductive age (15–49 years), and this group has been classified to include pregnant women, lactating women, lactating and pregnant women, and finally non-pregnant and non-lactating women. The SMS aimed to estimate the state of the main micronutrients such as iron, calcium and retinol (vitamin A) for different population groups of the targeted group.

A total of 8653 urinary iodine samples were collected from women in all 18 states. All women selected to provide a urine sample were asked to put the sample in a sterile, single-use plastic cup with a screw lid. All the samples from one cluster were packed in one carton, transported to the nearest suitable laboratory in each state and stored in a freezer kept at −20 °C until transport to NPHL to be analyzed for iodine. Urinary iodine was measured using ammonium persulfate. The results were obtained with a spectrophotometer, specifically spectroscopy model 40, which WHO recommends.

#### 2.1.2. Data Analysis

The full dataset was entered and arranged in a single Excel sheet. The R language for statistical computing v4.0.0 was used to perform the data analysis, and the R-analytic Flow (RAF) interactive development environment (IDE) was implemented.

#### 2.1.3. Definition of Indicators

1. Iron and C-reactive protein (CRP): the serum ferritin, and CRP were used to estimate the state of iron, the normal value is from 12 µg/L to 150 µg/L. The cut-offs of serum ferritin for children under the age of five years is <12 µg/L and for older than five years is <15 µg/L. There is an adjustment for the serum ferritin by 0.65 if the respondent was in an active inflammation process based on the values in Table 1.

2. Hemoglobin (Hb): various types of anemia were determined by measuring the concentration level of Hb; see Table 2. Hb values have been adjusted according to the altitude. The data of altitude were taken for every single PSUs from the Shuttle Radar Topography Mission (SRTM) by using a publicly available elevation model.

3. Iodine: Iodine deficiency was diagnosed at the population level based on median urinary iodine concentrations in populations of interest; see Table 3.

## 3. Results

### 3.1. Micronutrient Intake

#### 3.1.1. Vitamin-Rich Food Consumption

The level of consumption of vitamin A-rich foods for reproductive-age women was 67.36%, with the greater part coming from animal sources. Across states, there is great variability: Kassala state had the highest proportion of vitamin A-rich foods consumption at 88.28%. By contrast, East Darfur state had the lowest vitamin A-rich food consumption from any source of 34.50%. Child consumption of vitamin A-rich foods from any source was low at 23.44%. River Nile state had the highest consumption rate at 41.51%, while Blue Nile state had the lowest rate at 6.32% (see Table 4).

The consumption rate of vitamin B-rich foods among reproductive-age women was 62.13%; across the state level, only 33.3% of states had more than 70% of women consuming vitamin B-rich foods. Al-Gezira state had the highest proportion of vitamin B-rich food consumption at 78.40%. In comparison, East and North Darfur states had the lowest (25.05% and 26.82%, respectively). There was also an overall low child consumption of vitamin B-rich foods at 11.02%. Central Darfur state had the highest proportion of vitamin B-rich food consumption at 33.39%, while East Darfur and Kassala states had the lowest proportion at 1.39 and 2.39%, respectively.

#### 3.1.2. Minerals-Rich Food Consumption

Consumption of iron-rich foods among reproductive-age women (15–49 years of age) was at 67%. Across states, 44% had more than 70% of surveyed women who consumed iron-rich foods. Blue Nile and South Darfur had the highest proportion at 79% for both, while Red Sea state had the lowest proportion at 28%. Consumption of calcium-rich foods was relatively low among reproductive-age women (15–49 years of age) at 48%. Of all the states, only one had more than 70% of women consuming calcium-rich foods, which was a serving in the last 24 h. Kassala state had the highest proportion at 77%, followed by Al-Gezira at 62%, while South Kourdofan and Blue Nile states had the lowest at 22% and 19%, respectively. Consumption of zinc-rich foods among reproductive-age women (15–49 years of age) was 70%. Half of the state in the country had more than 70% of women who consumed zinc-rich foods. The highest proportion of zinc-rich food consumption was in Blue Nile state at 91%. In children, the proportion of iron-, calcium-, and zinc-rich foods consumption was low at 12%, 18%, and 15%, respectively. There was little variability across states; see Table 5.

### 3.2. Prevalence of Micronutrient Deficiency

#### 3.2.1. Iron Deficiency

Table 6 presents the prevalence of iron deficiency among women of reproductive age and children. Iron deficiency in non-pregnant women of reproductive age in Sudan was 47%, and the prevalence exceeded 20% in every state. Iron deficiency was a significant problem among pregnant women of reproductive age in Sudan with 58% of them nationally experiencing iron deficiency. Similarly, in children, the prevalence of iron deficiency was high at 54% nationally with a prevalence as high as 40% and above in 16 out of 18 states. West Darfur had the lowest iron deficiency prevalence among women (pregnant and non-pregnant) and children.

#### 3.2.2. Anemia

The prevalence of any anemia among women of reproductive age and children is shown in Table 7. The level of anemia among non-pregnant women of reproductive age for the whole of Sudan was high at 30%, and all 18 states had an anemia prevalence in non-pregnant women of reproductive age at or above 20%. The Red Sea had the highest prevalence at 46.36%, while Khartoum had the lowest prevalence at 19.88%. Similarly, the level of anemia among pregnant women was also high at 37%. In half of Sudan’s states, the prevalence of anemia among pregnant women of reproductive age exceeds 40%, East and central Darfur states had the highest prevalence at around 58.67% for both states. Among children, about 48% in Sudan suffer from some anemia. Among the states, East Darfur had the highest prevalence at 66.44%. Only one state, West Darfur, has a prevalence of anemia of less than 40%; however, the other 17 states has a prevalence in excess of 40%.

Based on the findings, the relative proportion of iron deficiency anemia (IDA) that is due to iron deficiency is likely to be less than half among children under five years, IDA was at 24% compare to iron deficiency prevalence of 54% nationally. Meanwhile, among women of reproductive age, IDA prevalence was 16% among non-pregnant and 21% among pregnant women compared to the iron deficiency prevalence of 47% among non-pregnant and 58% among pregnant women.

#### 3.2.3. Iodine Status

Generally, non-pregnant women of reproductive age in Sudan have adequate urinary iodine concentration at 108 μ/L, which indicates that the non-pregnant women of reproductive age in Sudan have an adequate iodine intake. However, this situation is heterogeneous across the states, with 7 out of the 18 states having an adequate urinary iodine concentration; Red Sea state has the highest concentration rate above requirements at 258 μ/L. The other 10 states have a median urinary iodine concentration lower than 100 μg/L. Five states have iodine deficiency between 50 and 99 μ/L, such as Al-Gadarif, North Kordofan, West Kordofan, Sennar, and South Darfur, and five other states have iodine deficiency below 50 μ/L; Central Darfur, East Darfur, Northern Darfur, West Darfur, and South Kordofan. On the other hand, the iodine status of lactating non-pregnant and pregnant women of reproductive age in Sudan is insufficient; across the states, for instance, the iodine status of lactating non-pregnant have insufficient iodine status (81.5 μ/L). Fourteen states have the insufficient iodine level <100 μ/L, East Darfur (10.4 μg/L) having the worst median urinary iodine concentration, while, Kassala, Khartoum, Northern and Red Sea states have an adequate concentration. Similarly, the iodine status of pregnant women of reproductive age in Sudan is insufficient at 101 μ/L. Findings show sixteen states have insufficient iodine status <150 μ/L, only in Khartoum and Red Sea states, pregnant women of reproductive age have an adequate iodine status (182 and 199 μ/L, respectively).

## 4. Discussion

This manuscript presents the micronutrient intake and prevalence among reproductive-age women (15–49 years of age) and their children in Sudan based on the simple spatial survey (S3M II). Knowing the intake and prevalence of micronutrients deficiency is crucial for determining the overall nutrition status of the population and tracking the progress of Sudan toward the Global Nutrition Targets with a focus on anemia. The findings provide information for health partners and policymakers about the population status of micronutrient indicators and the need for ongoing monitoring and evaluation to improve the nutrition of Sudan’s population to ensure evidence-based programming according to the priority areas.

The consumption of iron-rich foods among reproductive-age women (15–49 years of age) was relatively moderate at 66%; our observation regarding the intake of iron-rich foods among reproductive-age women is consistent with other studies conducted in Brazil [15]. However, the iron deficiency among women of reproductive age was high. The prevalence of iron deficiency was 47% among non-pregnant women of reproductive age and 58% among pregnant women; a similar study from Pakistan showed that 62% of women had a high prevalence of iron deficiency significantly associated with anemia [16]. This indicates that iron deficiency is common among women of reproductive age in Sudan as a whole and in all the states. These high prevalence rates were high enough to warrant being labeled as iron deficiency, a public health problem in Sudan. Community awareness interventions are highly encouraged to increase the iron and other minerals consumption from daily available food; such as meat, fish, eggs, cereals, local fruits and green leafy vegetables.

Various determinants of anemia have been recognized in the literature, including inadequate food intake, chronic disease, infections, poverty, high parity pregnancy, and poor access to health services, in addition to genetic and environmental factors [16,17,18,19,20]. The prevalence of all types of anemia among women of reproductive age was high. Among non-pregnant women, anemia prevalence was 30%. Anemia among pregnant women is also concerning: about 37% had anemia; this prevalence of anemia in pregnancy was consistent with data observed in two studies from Nigeria and Ethiopia [21,22]. Anemia prevalence among children is at critical levels; 48% of children had anemia, the findings of a high prevalence of anemia among children in this survey are similar to the findings of several studies in developing countries [23,24]. All 18 states had an anemia prevalence in women of reproductive age above 20%. In children, 17 states have a prevalence in excess of 40%. Consequently, each of those 17 states can be classified as experiencing levels of severe anemia that is of public health importance.

Reduction of anemia in women of reproductive age by 50% is a crucial target of the Global Nutrition Targets that should be achieved by the end of this decade [25,26]. Anemia among non-pregnant women is increased slightly from 29% in 2012 to 30% in 2019. Similarly, anemia among pregnant women increased to 37% in 2019 comparing to baseline values of 34% in 2012 [27]. Sudan still needs more efforts from the government and stakeholders to achieve the Global Nutrition Targets through implementing strategies, policies, and regulatory measures to address anemia and other forms of malnutrition.

Iodine deficiency is especially serious for pregnant and lactating women because of the negative consequences for both mother and infant. The median urinary iodine concentration (μg/L) of non-pregnant women of reproductive age in Sudan was adequate at 108 μg/L. The iodine status for non-pregnant lactating women is much worse. Nationally, the iodine status is suboptimal; likewise, the iodine status of pregnant women of reproductive age is inadequate. A high prevalence of iodine deficiency in Sudan indicates a critical issue; the salt iodization is not practiced equally across the country [28]. A study was revealed that iodine concentration in salt in Red Sea state was high at 150–360 mg iodine (KOI3)/kg salt, whereas those from White Nile State had low levels to detect; the same study showed that consumption of Nile fish might have provided sufficient iodine [29]. This will not only affect women but also children; iodine deficiency is widespread among pregnant women despite the implementation of iodine fortification interventions in Africa [1].

## 5. Conclusions

Micronutrient deficiency is considered to be a public health problem in Sudan that needs to be improved. Anemia in women of reproductive age and children showed high prevalence. The situation was also similar to iron deficiency; it is considered a crucial problem in women of reproductive age, especially pregnant women and children. Therefore, interventions that aim to improve the status of micronutrients among women and children are highly recommended; Sudan needs more efforts to create an enabling environment through legislation, policies, and strategies to strengthen the nutrition-sensitive and specific interventions and to improve the status of micronutrients among women and children, to focus on food fortification, micronutrients supplements, and counseling on micronutrients intake for mothers during antenatal and postnatal services, as well as raising community awareness about the importance of micronutrients intake for women and children. Improving access to safe drinking water, malaria control, and deworming will contribute to the overall improvement of micronutrient status in the community. Stakeholders including the government should take into consideration the variation in prevalence of micronutrients deficiencies and culture differences in terms of food habits between states and localities to prioritize the areas during planning and implementation of nutrition programs.

## Figures and Tables

**Table 1:** Cut-off of Iron and C-reactive protein.

Relative Extent of Iron Stores on the Basis of Serum Ferritin Concentration (μg/L)				
	**Serum Ferritin (μg/L)**			
	**Less than 5 Years**		**5 Years or Older**	
	**Male**	**Female**	**Male**	**Female**
Depleted iron stores	<12	<12	<15	<15
Depleted iron stores in the presence of infection	<30	<30		
Severe risk of iron overload (adults)			>200	>150
Cut-offs to determine acute inflammation				
**Acute phase protein**				
Cut-off	CRP		AGP	
	>5 mg/L		>1 g/L	

Source: World Health Organization, ‘Haemoglobin Concentrations for the Diagnosis of Anaemia and Assessment of Severity’, Vitamin and Mineral Nutrition Information System, Geneva, World Health Organization, 2011, pp. 1–6, www.who.int/vmnis/indicators/haemoglobin.pdf, (accessed 12 July 2021).

**Table 2:** Hemoglobin levels to diagnose anemia at sea level (g/L).

Population	Hb Concentration (g/L)
Children 6–59 months	<110
Children 5–11 years	<115
Children 12–14 years	<119
Non-pregnant women (15 years and above)	<119
Pregnant women	<110

Source: World Health Organization, ‘Haemoglobin Concentrations for the Diagnosis of Anaemia and Assessment of Severity’, Vitamin and Mineral Nutrition Information System, Geneva, World Health Organization, 2011, pp. 1–6, www.who.int/vmnis/indicators/haemoglobin.pdf, (accessed 12 July 2021).

**Table 3:** Epidemiologic criteria for assessing population-level iodine nutrition based on median urinary iodine concentrations in different population groups.

Median Urinary Iodine Concentration (μg/L)	Indication of Population Iodine Intake
Pregnant women	
<150	Insufficient
150–249	Adequate
250–499	Above requirements
≥500	Excessive
Lactating women and children under 2 years of age	
<100	Insufficient
≥100	Adequate

Source: Centers for Disease Control and Prevention, World Health Organization, Nutrition International, UNICEF. Micronutrient survey manual. Geneva: World Health Organization; 2020.

**Table 4:** Consumption of vitamins-rich foods among reproductive-age women (15–49 years of age) and their children.

State	Reproductive-Age Women						Children under Five					
	Vitamin A-Rich Foods			Vitamin B-Rich Foods (B1/B2/B3/B6/B12)			Vitamin A-Rich Foods			Vitamin B-Rich Foods (B1/B2/B3/B6/B12)		
	Estimati on	95% LCL	95% UCL	Estimati on	95% LCL	95% UCL	Estimati on	95% LCL	95% UCL	Estimati on	95% LCL	95% UCL
Al Gedaref	62.01	52.02	72.06	56.28	442.80	679.75	18.13	0.01	49.54	12.15	0.01	34.78
Al- Gezira	73.86	63.42	84.30	78.40	70.56	86.23	21.73	0.01	46.78	11.53	0.01	30.81
Blue Nile	43.88	34.30	53.46	75.51	66.93	84.09	6.32	3.78	19.19	10.86	0.01	24.55
Central Darfur	50.98	39.00	62.97	72.81	63.40	82.22	29.02	27.82	48.63	33.39	9.47	57.31
East Darfur	34.50	25.89	43.10	25.05	17.67	32.44	21.47	15.46	41.81	1.39	0.01	14.67
Kassala	88.28	82.59	93.96	49.23	40.57	57.88	14.93	12.34	25.46	2.39	0.37	5.61
Khartoum	76.43	68.83	84.04	76.38	68.70	84.07	31.27	27.43	48.41	15.15	0.01	36.56
North Darfur	61.10	50.22	71.98	26. 82	17.84	37.81	34.65	19.95	36.47	8.35	0.01	28.02
North Kordofan	63.71	54.83	72.60	67.47	59.54	75.39	16.14	10.85	20.54	13.45	1.87	25.03
Northern	67.09	57.52	76.67	41.68	28.97	54.39	30.74	16.75	35.49	6.02	0.01	26.35
Red Sea	65.95	57.26	74.64	37.53	27.71	47.35	18.97	13.91	33.03	4.22	0.01	19.97
River Nile	79.39	72.54	86.23	53.98	45.43	62.53	41.51	30.61	44.47	5.82	0.01	14.65
Sennar	62.92	52.84	73.04	76.47	68.95	83.99	20.41	9.43	21.71	8.84	0.01	24.46
South Darfur	74.87	64.04	85.70	55.39	53.16	57.77	27.97	12.36	20.88	11.81	0.01	38.81
South Kordofan	38.47	28.23	34.12	65.70	56.31	75.10	13.99	9.52	19.52	12.12	0.01	29.47
West Darfur	55.98	45.74	66.22	68.76	58.77	78.74	15.39	5.88	19.86	14.06	0.01	39.02
West Kordofan	61.24	51.50	70.97	53.62	43.57	63.67	19.91	11.37	20.25	7.73	0.01	30.40
White Nile	74.68	67.41	81.95	76.16	70.17	82.15	23.44	12.60	27.59	15.32	0.01	34.83
Sudan	67.36	58.07	76.65	62.13	53.13	71.13	23.44	15.66	16.03	11.02	0.01	30.41

LCL and UCL: Lower and Upper Confidence Interval Limits.

**Table 5:** Consumption of minerals-rich foods among reproductive-age women (15–49 years of age) and their children.

State	Reproductive-Age Women									Children under Five								
	Iron-Rich Foods			Calcium-Rich Foods			Zinc-Rich Foods			Iron-Rich Foods			Calcium-Rich Foods			Zinc-Rich Foods		
	Estimati on	95% LCL	95% UCL	Estimati on	95% LCL	95% UCL	Estimati on	95% LCL	95% UCL	Estimati on	95% LCL	95% UCL	Estimati on	95% LCL	95% UCL	Estimati on	95% LCL	95% UCL
Al Gedaref	69.27	60.17	78.38	34.73	23.08	46.39	63.50	53.35	73.66	14.99	0.01	38.99	7.77	0.01	36.29	12.81	0.01	35.46
Al- Gezira	77.77	70.27	85.26	62.26	50.20	74.32	85.96	79.48	92.44	13.21	0.01	33.43	19.83	0.01	44.23	16.85	0.01	40.36
Blue Nile	79.64	70.30	88.98	18.69	10.76	26.61	90.85	83.86	97.85	10.62	0.01	26.26	4.42	0.01	24.34	28.69	7.91	49.47
Central Darfur	72.96	62.72	83.19	24.31	13.03	35.60	77.54	68.82	86.27	17.91	0.01	44.16	20.20	0.01	52.67	26.04	1.94	50.15
East Darfur	33.35	24.82	41.88	28.42	20.40	36.44	28.53	20.73	36.33	3.74	0.01	27.09	20.63	0.01	49.07	1.39	0.00	14.46
Kassala	43.26	32.43	54.08	76.69	71.79	87.58	54.75	45.75	63.76	1.55	0.01	16.75	14.31	0.01	40.79	1.56	0.01	14.48
Khartoum	72.39	63.88	80.90	51.97	41.58	62.37	84.07	78.03	90.11	14.96	0.01	34.70	21.31	0.01	45.50	26.44	3.87	49.00
North Darfur	55.21	43.42	67.00	27.50	17.73	37.27	33.75	24.40	43.09	24.08	1.63	46.53	14.46	0.01	37.41	9.24	0.01	29.65
North Kordofan	69.30	60.15	78.46	45.48	37.22	53.74	80.21	72.84	87.58	14.45	1.99	26.91	11.75	0.01	25.02	14.95	3.31	26.59
Northern	54.84	45.13	64.55	28.61	15.99	41.24	53.12	39.69	66.56	3.61	1.01	12.50	20.51	0.01	48.38	6.86	0.01	26.55
Red Sea	28.04	17.36	38.71	57.65	47.62	67.67	47.15	37.26	57.03	0.59	0.01	5.47	19.03	0.01	38.60	4.93	0.01	22.14
River Nile	63.93	55.55	72.31	61.81	53.86	69.77	62.51	53.58	71.45	5.01	0.01	13.80	38.44	15.21	61.68	9.83	0.51	19.09
Sennar	76.68	69.24	84.12	49.63	37.57	61.69	82.52	75.67	89.37	7.90	0.01	22.54	18.67	0.01	39.18	12.89	1.50	24.28
South Darfur	79.46	69.15	89.77	47.12	33.19	61.05	64.89	54.18	75.50	13.75	0.01	30.60	24.96	0.01	72.06	12.89	1.50	24.28
South Kordofan	66.72	57.59	75.86	21.75	13.51	29.98	85.96	79.16	92.76	11.38	0.01	28.04	11.40	0.01	26.45	19.78	0.38	39.18
West Darfur	72.61	63.62	81.61	38.51	27.73	49.30	71.30	60.89	81.70	14.41	0.01	40.71	11.11	0.01	36.62	15.55	0.01	42.02
West Kordofan	63.60	54.09	73.11	46.45	35.34	57.56	57.27	47.11	67.43	9.82	0.01	31.93	12.81	0.01	35.67	8.41	0.01	31.56
White Nile	77.95	71.86	84.04	59.79	51.50	68.09	83.36	77.74	88.98	15.53	0.01	34.18	11.40	0.01	33.46	18.09	0.01	37.96
Sudan	66.75	57.61	75.89	47.69	37.12	58.26	69.72	61.40	78.04	12.28	0.01	31.05	17.62	0.01	44.07	14.99	0.01	34.51

LCL and UCL: Lower and Upper Confidence Interval Limits.

**Table 6:** The estimation of median-adjusted serum ferritin (ng/mL), iron deficiency among reproductive-age women (15-49 years of age), and these women’s children.

States	Non-Pregnant Women			Pregnant Women			Children under Five		
	Iron Deficiency			Iron Deficiency			Iron Deficiency		
	Estimate	95%.LCL	95%.UCL	Estimate	95%.LCL	95%.UCL	Estimate	95%.LCL	95%.UCL
Al-Gadarif State	36.93	30.43	44.57	69.05	55.22	82.72	49.08	41.36	56.60
Al-Gazeera State	46.12	36.61	54.73	54.10	41.06	67.96	56.87	47.26	66.80
Blue Nile State	36.30	27.53	47.81	45.45	31.51	62.72	37.04	29.09	46.72
Central Darfur State	32.89	24.11	41.07	76.19	53.34	98.17	44.93	37.02	54.33
East Darfur State	49.03	40.66	57.48	69.35	55.14	88.11	44.59	37.87	51.86
Kassala State	56.34	47.42	64.47	75.44	60.65	88.68	67.71	58.67	75.87
Khartoum State	46.75	38.10	55.32	57.35	43.02	71.65	60.24	53.05	67.44
North Darfur State	40.39	33.62	48.64	52.81	37.76	68.44	46.99	40.00	53.29
North Kourdofan State	51.33	43.07	60.05	62.30	49.52	75.44	65.29	57.74	72.30
Northern State	42.06	33.11	51.21	65.33	50.74	77.23	59.01	51.83	65.76
Red Sea State	63.76	55.55	72.07	64.58	44.99	78.96	67.93	58.54	74.75
River Nile State	54.65	46.75	62.11	56.72	41.51	71.93	62.54	54.89	69.23
Sennar State	43.73	35.24	52.73	63.44	51.91	77.57	52.36	43.75	60.74
South Darfur State	58.26	47.98	71.08	54.17	38.20	72.10	40.31	32.57	47.32
South Kourdofan State	42.37	34.97	51.00	67.31	49.98	87.26	51.47	44.30	60.00
West Darfur State	28.25	22.45	35.67	25.45	13.68	41.07	28.60	23.18	34.19
West Kourdofan State	38.36	29.35	50.66	51.72	41.81	63.97	40.51	32.19	47.36
White Nile State	46.54	39.32	54.17	53.17	42.13	66.45	51.77	45.21	59.03
Sudan	47.07	38.21	55.92	58.44	43.55	73.32	53.55	45.96	61.14

LCL and UCL: Lower and Upper Confidence Interval Limits.

**Table 7:** An estimation of median adjusted serum hemoglobin concentration (g/dL) and prevalence of any anemia (%) among reproductive-age women (15–49 years of age) and their children.

States	Non-Pregnant Women			Pregnant Women			Children under Five		
	anemia			Anemia			Anemia		
	Estimate	95%.LCL	95%.UCL	Estimate	95%.LCL	95%.UCL	Estimate	95%.LCL	95%.UCL
Al-Gadarif State	30.27	23.48	38.09	28.24	16.84	40.26	50.92	42.87	57.34
Al-Gazeera State	25.94	19.59	34.09	34.44	24.65	45.97	47.81	40.81	54.93
Blue Nile State	37.79	30.00	45.94	51.61	38.75	65.32	60.93	55.09	66.43
Central Darfur State	36.36	29.09	44.96	58.33	46.82	71.26	45.25	37.32	54.45
East Darfur State	40.28	33.16	47.68	58.67	43.92	75.26	66.44	60.05	73.71
Kassala State	39.25	32.37	47.64	46.05	31.73	62.94	52.82	47.53	58.67
Khartoum State	19.88	14.23	26.20	26.19	12.33	40.88	44.84	36.21	53.60
North Darfur State	37.18	29.56	43.63	52.22	39.64	64.97	46.68	38.57	53.08
North Kourdofan State	30.58	24.93	36.24	38.67	27.13	51.68	51.49	45.71	56.92
Northern State	21.90	16.44	27.73	32.91	19.70	47.83	44.69	37.49	51.53
Red Sea State	46.36	39.43	53.37	44.00	28.19	61.79	54.96	46.96	61.25
River Nile State	34.48	27.12	43.92	23.19	11.49	39.40	55.58	47.75	62.18
Sennar State	31.73	25.73	38.77	25.21	16.03	35.12	50.15	44.76	55.52
South Darfur State	31.87	24.72	40.28	41.56	28.56	54.13	41.44	34.35	48.91
South Kourdofan State	29.36	23.56	36.10	51.88	39.10	64.77	48.88	42.56	55.17
West Darfur State	24.66	18.18	31.48	25.88	13.84	39.80	32.99	28.09	37.73
West Kourdofan State	24.61	17.47	32.52	35.40	24.03	49.23	40.20	32.38	48.33
White Nile State	22.41	17.00	28.65	22.22	13.83	31.22	44.19	37.26	50.95
Sudan	29.66	22.62	36.69	36.61	23.89	49.32	48.09	41.08	55.09

LCL and UCL: Lower and Upper Confidence Interval Limits.

## Data Availability

Restrictions apply to the availability of these data. Data was obtained from Ministry of Health and are available [from the first author] with the permission of the Ministry of Health.

## References

[1] Nunn R.L., Kehoe S.H., Chopra H., Sahariah S.A., Gandhi M., Di Gravio C., Coakley P.J., Cox V.A., Sane H., Shivshankaran D. (2019). Dietary micronutrient intakes among women of reproductive age in Mumbai slums. Eur. J. Clin. Nutr..

[2] UNICEF (2019). The State of the World’s Children Report 2019. Children Food and Nutrition: Growing Well in a Changing World.

[3] WHO, WFP, UNICEF (2007). Preventing and Controlling Micronutrient Deficiencies in Populations Affected by an Emergency.

[4] Bailey R.L., West K.P., Black R.E. (2015). The Epidemiology of Global Micronutrient Deficiencies. Ann. Nutr. Metab..

[5] Vaivada T., Gaffey M.F., Das J.K., Bhutta Z.A. (2017). Evidence-based interventions for improvement of maternal and child nutrition in low-income settings: What’s new?. Curr. Opin. Clin. Nutr. Metab. Care.

[6] Smith E.R., Shankar A.H., Wu L.S., Aboud S., Adu-Afarwuah S., Ali H., Agustina R., Arifeen S., Ashorn P., Bhutta Z.A. (2017). Modifiers of the effect of maternal multiple micronutrient supplementation on stillbirth, birth outcomes, and infant mortality: A meta-analysis of individual patient data from 17 randomised trials in low-income and middle-income countries. Lancet Glob. Health.

[7] Stevens G.A., Finucane M.M., De-Regil L.M., Paciorek C.J., Flaxman S.R., Branca F., Peña-Rosas J.P., Bhutta Z.A., Ezzati M. (2013). Global, regional, and national trends in haemoglobin concentration and prevalence of total and severe anaemia in children and pregnant and non-pregnant women for 1995–2011: A systematic analysis of population-representative data. Lancet Glob. Health.

[8] Devakumar D., Fall C.H., Sachdev H.S., Margetts B.M., Osmond C., Wells J.C., Costello A., Osrin D. (2016). Maternal antenatal multiple micronutrient supplementation for long-term health benefits in children: A systematic review and meta-analysis. BMC Med..

[9] Abu-Manga M., Al-Jawaldeh A., Qureshi A.B., Ali A.M.E., Pizzol D., Dureab F. (2021). Nutrition Assessment of Under-Five Children in Sudan: Tracking the Achievement of the Global Nutrition Targets. Children.

[10] Osendarp S.J.M., Brown K.H., Neufeld L.M., Udomkesmalee E., Moore S.E. (2020). The double burden of malnutrition—Further perspective. Lancet.

[11] Shommo S.A., Zumrawi F.Y., Saeed A.M. (2015). Micronutrients Dietary Intakes of Pregnant Sudanese Women. Int. J. Sci. Res..

[12] Nyuar K.B., Khalil A.K., Crawford M.A. (2012). Dietary intake of Sudanese women: A comparative assessment of nutrient intake of displaced and non-displaced women. Nutr. Health.

[13] FMOH (2020). Simple Spatial Survey Method (S3M II) Sudan Micronutrient Survey Report. SNational Nutrition Program.

[14] FMOH (2018). Simple Spatial Survey Method (S3M II) for Sudan—2018. https://data.humdata.org/dataset/simple-spatial-survey-method-s3m-ii-for-sudan-2018.

[15] Sato A.P.S., Fujimori E., Szarfarc S.C., Borges A.L.V., Tsunechiro M.A. (2010). Food Consumption and Iron Intake of Pregnant and Reproductive Aged Women. Rev. Lat. Am. De Enferm..

[16] Ali S.A., Abbasi Z., Shahid B., Moin G., Hambidge K.M., Krebs N.F., Westcott J.E., McClure E.M., Goldenberg R.L., Saleem S. (2020). Prevalence and determinants of anemia among women of reproductive age in Thatta Pakistan: Findings from a cross-sectional study. PLoS ONE.

[17] Dasgupta A., Sarkar K., Chowdhury R., Ray A., Shahbabu B. (2016). Anemia and its determinants among women of reproductive age of a slum in Kolkata: A focus group discussion among health workers in a slum of Kolkata. J. Fam. Med. Prim. Care.

[18] Mawani M., Ali S.A., Bano G., Ali S.A. (2016). Iron Deficiency Anemia among Women of Reproductive Age, an Important Public Health Problem: Situation Analysis. Reprod. Syst. Sex Disord..

[19] Jamnok J., Sanchaisuriya K., Sanchaisuriya P., Fucharoen G., Fucharoen S., Ahmed F. (2020). Factors associated with anaemia and iron deficiency among women of reproductive age in Northeast Thailand: A cross-sectional study. BMC Public Health.

[20] Nguyen P.H., Gonzalez-Casanova I., Nguyen H., Pham H., Truong T.V., Nguyen S., Martorell R., Ramakrishnan U. (2015). Multicausal etiology of anemia among women of reproductive age in Vietnam. Eur. J. Clin. Nutr..

[21] Dim C.C., Onah H.E. (2007). The prevalence of anemia among pregnant women at booking in Enugu, South Eastern Nigeria. Medscape Gen. Med..

[22] Addis Alene K., Mohamed Dohe A. (2014). Prevalence of Anemia and Associated Factors among Pregnant Women in an Urban Area of Eastern Ethiopia. Anemia.

[23] Bradley L. (2011). In All Candour: Taking Off the Mask. Int. J. Nutr. Pharmacol. Neurol. Dis..

[24] Foote E.M., Sullivan K.M., Ruth L.J., Oremo J., Sadumah I., Williams T.N., Suchdev P.S. (2013). Determinants of Anemia among Preschool Children in Rural, Western Kenya. Am. Soc. Trop. Med. Hyg..

[25] Hawkes C., Ruel M.T., Salm L., Sinclair B., Branca F. (2020). Double-duty actions: Seizing programme and policy opportunities to address malnutrition in all its forms. Lancet.

[26] WHO (2014). Global Nutrition Targets 2025: Policy Brief Series. https://www.who.int/publications/i/item/WHO-NMH-NHD-14.2.

[27] WHO The Global Nutrition Targets: Tracking Tool. 50% Reduction of Anemia in Women of Reproductive Age 2021. https://extranet.who.int/nhdtargets/en/Anaemia.

[28] Mahfouz M.S., Gaffar A.M., Bani I.A. (2012). Iodized salt consumption in Sudan: Present status and future directions. J. Health Popul. Nutr..

[29] Hussein I., Min Y., Ghebremeskel K., Gaffar A. (2012). Iodine status and fish intake of Sudanese schoolchildren living in the Red Sea and White Nile regions. Public Health Nutr..

